# A Heavy Metal Ion Water Quality Detection Model Based on Spectral Analysis: New Methods for Enhancing Detection Speed and Visible Spectral Denoising

**DOI:** 10.3390/s25072318

**Published:** 2025-04-05

**Authors:** Bingyang Sun, Shunsheng Yang, Xu Cheng

**Affiliations:** 1 School of Civil Engineering, Southwest Jiaotong University, Chengdu 610031, China; s20017300005@my.swjtu.edu.cn (B.S.); yss@swjtu.edu.cn (S.Y.); 2 College of Railway and Electrical Engineering, Sichuan Railway College, Chengdu 610097, China; 3 School of Civil Engineering, Chongqing University, Chongqing 400045, China

**Keywords:** spectral signal-to-noise ratio, water quality detection, spectral analysis, Singular Value Decomposition, Convolutional Neural Network

## Abstract

This paper analyzes the current state of water quality detection equipment and, based on the demand for portable water quality detection systems that are on-site, rapid, accurate, cost-effective, and capable of multi-parameter measurements using spectral analysis, represents the future development direction of water quality detection. By focusing on indicators of heavy metal ion water pollution, this study aims to achieve the “rapid and accurate detection of water quality using spectral analysis” and emphasizes key technologies such as “visible absorption spectroscopy in photoelectric detection technology and spectral analysis”, “spectral denoising methods”, and “Convolutional Neural Network (CNN) modeling and deployment”. A novel combined denoising method integrating Ensemble Empirical Mode Decomposition (EEMD) and Singular Value Decomposition (SVD) is developed and applied for the first time in spectral water quality detection to improve accuracy. The system uses a ZYNQ-based spectral analysis platform to detect heavy metal ion concentrations, enhancing detection speed. Comparative tests with copper ion standard solutions against Chinese national standards show good accuracy and reproducibility. The developed EEMD-SVD method demonstrates superior denoising effectiveness in processing actual spectral data within the water quality detection system.

## 1. Introduction

In the 21st century, water pollution has become a global challenge that profoundly affects human survival and development [1]. Research indicates that water pollution not only poses a serious threat to human health and even endangers life safety, but also causes an undeniable hindrance to the sustainable development of society and the economy [2]. Among these pollutants, heavy metal ions, which are highly toxic and mobile, are particularly alarming [3]. For instance, the average concentrations of heavy metal elements such as chromium (Cr), cobalt (Co), iron (Fe), and nickel (Ni) in surface water bodies often significantly exceed the safety standards for drinking water. This not only poses a significant challenge to drinking water safety, but may also further exacerbate harm to human health through bioaccumulation in the food chain [4,5,6]. Therefore, timely and accurate water quality detection is of great significance for safeguarding health and protecting the environment.

The spectral analysis technique for water quality detection is one of the most commonly used methods in the field of water quality monitoring [7]. However, when employing spectral analysis for water quality detection, there are often many factors unrelated to the parameters being measured that interfere with data acquisition. For example, electromagnetic interference, inherent random noise from the instruments, white noise, constant-frequency noise, and high-frequency noise can all affect the spectral data on water quality, leading to baseline shifts and unstable signal fluctuations, thereby compromising the accuracy of the spectral data [8]. Specifically, these noise interferences reduce the signal-to-noise ratio (SNR), which poses significant challenges for the subsequent modeling and analysis of water quality parameters [9]. In the research on spectral noise reduction, different denoising methods are suitable for removing noise from different types of signals. The Gaussian Smoothing Filter (GS) can effectively reduce noise, but it may lead to loss of signal details. The Fast Fourier Transform (FFT) is effective for noise reduction in stationary signals. However, its capability in processing non-stationary signals is limited. Moreover, modifications to the signal amplitude via the FFT may affect the accuracy of spectral reconstruction. This method relies on good signal conditions and struggles to cope with complex water quality monitoring scenarios [10,11,12]. In numerous studies, the wavelet threshold method has been proven to effectively reduce noise while preserving signal features [13,14]. However, this method is sensitive to the choice of wavelet basis functions and the number of decomposition levels, and it requires careful parameter selection for optimal performance [15,16]. Empirical Mode Decomposition (EMD) and its extended version, Ensemble Empirical Mode Decomposition (EEMD), are adaptive signal processing techniques that have demonstrated effective capabilities in handling non-linear and non-stationary signals, showing good noise reduction performance. However, in practical applications, these methods may suffer from mode mixing, which can affect the accuracy of the signal [17,18]. Additionally, some studies have adopted hybrid denoising approaches to leverage the strengths of different methods, aiming to achieve breakthroughs in the precision and effectiveness of signal processing [19,20]. Yet, these hybrid methods still face issues such as low detection efficiency. In summary, existing spectral denoising techniques still have many limitations, especially in terms of automation and adaptability for heavy metal ion detection, where relevant research is still lacking.

To effectively eliminate invalid interferences in spectra while preserving waveform information to the greatest extent, this study proposes a novel spectral denoising method for the water quality detection of heavy metal ions, combining Ensemble Empirical Mode Decomposition (EEMD) and Singular Value Decomposition (SVD). This method takes into account the non-stationary and stochastic characteristics of visible spectra, and is capable of addressing various types of non-stationary noises. In addition, by constructing a water quality detection system, the challenges of transitioning from laboratory to outdoor online measurements have been overcome, achieving miniaturization, high efficiency, and high-precision measurements. The remainder of this paper is organized as follows. Section 2 introduces the proposed method and the relevant theories. Section 3 presents the constructed water quality detection system. Section 4 conducts field tests using copper ions as the target analyte to validate the proposed method and the monitoring system. Finally, Section 5 summarizes the main conclusions obtained in this study.

## 2. Simulation Modeling of the Water Quality Detection System

**Figure 1:**
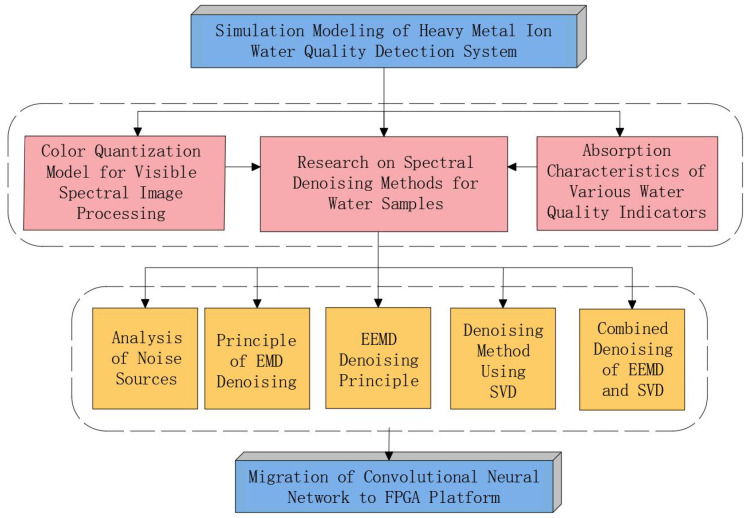
Procedure of the water quality detection system.

The technical roadmap for the simulation modeling of the water quality detection system is shown in Figure 1. This method is based on the color quantization model of visible spectral image processing and the absorption characteristics of various water quality indicators, and it focuses on research on spectral denoising methods for water samples. Based on the analysis of noise sources, the principles of denoising using Empirical Mode Decomposition (EMD), Ensemble Empirical Mode Decomposition (EEMD), and Singular Value Decomposition (SVD), a combined denoising method integrating EEMD and SVD was developed. This method was applied for the first time in spectral water quality detection. Finally, a Convolutional Neural Network (CNN) model was constructed and deployed on an FPGA platform to form a water spectral analysis system. The FPGA used was the Xilinx Zynq UltraScale+ MPSoC XCZU19EG, a powerful development board that integrates ARM Cortex-A53 and Cortex-R5 processing cores with programmable logic (FPGA) resources, suitable for complex system design and development. This approach successfully enabled the rapid and accurate detection of five heavy metal ions (arsenic (As), copper (Cu), hexavalent chromium (Cr), lead (Pb), and mercury (Hg)) in water pollution.

### 2.1. Visible Spectroscopy

When exposed to continuous visible electromagnetic waves in the range of 400 to 800 nanometers, sample molecules can absorb light and produce a specific spectrum known as the visible absorption spectrum, which is a type of electronic spectrum. The formation of the visible absorption spectrum is due to the electronic transitions within the molecules, accompanied by corresponding changes in vibrational and rotational energy levels. These changes result in broad and relatively simple peak shapes in the visible absorption spectrum, making it highly suitable for both qualitative and quantitative analysis. Each compound has a unique spectral fingerprint, and variations in sample concentration lead to differences in the absorbance intensity of the spectrum. Therefore, spectroscopy is considered an efficient method for detecting the composition and concentration of substances.

### 2.2. Absorption Characteristics of Water Quality Indicators

The absorption characteristics of the same substance are fixed, and each substance has its characteristic wavelength for light absorption. In water quality analysis, changes in the concentration of metal ions only alter the absorbance at the characteristic wavelength, without changing the wavelength itself. Therefore, it is feasible to analyze the concentration of different metal ions in water samples by measuring their absorption characteristics.

(1) Copper Ions: When copper ions react with the color developer (dibenzoyl-dioxime), a blue complex is formed. According to the principle of complementary colors, the complex absorbs yellow light. The characteristic absorption wavelength for copper ions is typically measured at 610 nm;

(2) Hexavalent Chromium Ions: When hexavalent chromium ions react with the color developer (diphenylcarbazide), a purple–red complex is formed. According to the principle of complementary colors, the solution absorbs green light at this wavelength. The characteristic absorption wavelength for hexavalent chromium ions is typically measured at 540 nm;

(3) Arsenic Ions: When arsenic ions react with the color developer (chromic acid method), a yellow complex is formed. According to the principle of complementary colors, the solution absorbs green light at this wavelength. The characteristic absorption wavelength for arsenic ions is typically measured at 540 nm;

(4) Lead Ions: When lead ions react with the color developer (dithizone), a red complex is formed. According to the principle of complementary colors, the solution absorbs green light at this wavelength. The characteristic absorption wavelength for lead ions is typically measured at 510 nm;

(5) Mercury Ions: When mercury ions react with the color developer (4-nitro-4′-fluorophenyl diazodiaminobenzene), a red complex is formed. According to the principle of complementary colors, the solution absorbs cyan light at this wavelength. The characteristic absorption wavelength for mercury ions is typically measured at 485 nm.

### 2.3. Common Color Quantization Models and Denoising Methods in Image Processing

Color quantization models are mathematical models that describe colors using a set of numerical values. The correct choice of model is beneficial for image processing and includes models such as the RGB quantization model, the HSV quantization model, and the grayscale quantization model. In image preprocessing, one of the most fundamental and important methods is image filtering. Image filtering can effectively eliminate random noise, Gaussian noise, and salt-and-pepper noise that may arise from measurement imaging or environmental factors.

### 2.4. Principle of EEMD-SVD Spectral Denoising Method

#### 2.4.1. Principle of EMD Denoising

Empirical Mode Decomposition (EMD) is an adaptive decomposition technique designed to decompose complex signals into a finite number of independent Intrinsic Mode Functions (IMFs). EMD is widely used for analyzing non-linear and non-stationary signals [21,22,23]. These IMFs exhibit similar characteristics in terms of extrema and zero-crossings. Specifically, between each pair of adjacent zero-crossings, there is an extremum, and the upper and lower envelopes show local symmetry along the time axis. The original signal can be reconstructed through the decomposition of the signal as follows:

(1) $$x(t)=\sum_{i=1}^{n}c_{i}(t)+r_{n}(t)$$

where *c_i_*(*t*) represents the *i*-th IMF and *r_n_*(*t*) is the residual term.

Through EMD, the obtained multiple IMF components are categorized into effective and ineffective components. It is essential to thoroughly eliminate the ineffective components to accurately reflect the characteristics of the visible light spectrum. To optimize the selection of IMF components, the variance contribution rate and Pearson correlation coefficient are introduced [24,25].

The Pearson correlation coefficient: This coefficient is used to quantify the similarity between the IMF components of the visible light spectrum and the original signal obtained through EMD. The calculation formula can be expressed as follows:

(2) $$\rho (x,c_{i})=\frac{\sum_{i=1}^{N}\left[x(t)-\bar{x}][c_{i}(t)-{\bar{c}}_{i}\right]}{\sqrt{\sum_{i=1}^{N}{\left[x(t)-\bar{x}\right]}^{2}\sum_{i=1}^{N}{\left[c_{i}(t)-{\bar{c}}_{i}\right]}^{2}}}$$

In the formula, x(t) represents the visible light spectrum, while $\bar{x}$ and ${\bar{c}}_{i}$ refer to the arithmetic means of x(t) and $c_{i}(t)$, respectively; $\rho (x,c_{i})$ is the correlation coefficient between the *i*-th IMF component and the original signal x(t), and *N* represents the length of the signal.

Variance Proportion *v*: This metric aims to assess the variance of each IMF component in the original signal, and can be expressed as follows:

(3) $$\nu (i)=\frac{\frac{1}{N}\sum_{i=1}^{N}{\left[c_{i}(t)\right]}^{2}-{\left[\frac{1}{N}\sum_{i=1}^{N}c_{i}(t)\right]}^{2}}{\sum_{t=1}^{M}\left\{\frac{1}{N}\sum_{i=1}^{N}{[c_{i}(t)]}^{2}-{\left[\frac{1}{N}\sum_{i=1}^{N}c_{i}(t)\right]}^{2}\right\}}$$

In the formula, *M* represents the total number of IMF components obtained from the EMD of the original signal and ν(i) can describe the magnitude of the contribution of the *i*-th IMF component $c_{i}(t)$ to the original signal x(t).

#### 2.4.2. Principle of EEMD Denoising

Empirical Mode Decomposition (EMD) is an adaptive decomposition technique specifically designed for non-linear and time-varying signals. However, it has a major issue—mode mixing—that can degrade the accuracy of the decomposition results [26]. Mode mixing refers to the phenomenon where different Intrinsic Mode Functions (IMFs) influence each other during the EMD process, making it difficult to clearly separate the components of the original signal.

To address this issue, Huang et al. proposed the Ensemble Empirical Mode Decomposition (EEMD) method [27,28]. This method enhances the effectiveness of EMD by adding Gaussian white noise to the original signal multiple times [27,29]. This process, which introduces randomness, helps to smooth the effects of singularities, maintain signal continuity, and effectively prevent mode mixing [17]. The EMD process is then applied to the noise-added signals to obtain multiple sets of Intrinsic Mode Functions (IMFs) [30]. Finally, by averaging these IMFs, a set of more accurate and physically meaningful IMFs is obtained. The specific process is as follows:

1. Add a random Gaussian white noise sequence n(t) of the same length to the original signal x(t), ensuring that the added noise n(t) maintains the variance and has a mean value of zero. This will result in a signal sequence $x^{\prime }(t)$ that contains white noise, as shown below:
  (4) $$x^{\prime }(t)=x(t)+n(t)$$
2. Using the EMD theory to decompose $x^{\prime }(t)$, a set of Intrinsic Mode Functions (IMFs) and $r_{c}(t)$ (the residual component) will be generated, as shown in (5).
  (5) $$x^{\prime }(t)=\sum_{j=1}^{n}IMF_{j}(t)+r_{c}(t),j=1,2,3\ldots ,n$$
3. Repeat the previous two steps to obtain m instances of $x^{\prime }(t)$, as shown in (6).
  (6) $$x^{\prime }(t)=\sum_{j=1}^{n}{IMF}_{i,j}(t)+r_{c,j}(t),i=1,2,3\ldots ,m$$
4. Divide the average of all the IMFs by the added white noise from $r_{c}(t)$ to obtain the final results, as shown in (7) and (8).
  (7) $$IMF_{j}(t)=\frac{1}{m}\sum_{j=1}^{m}{IMF}_{i,j}(t),j=1,2,3,\ldots ,n$$
  (8) $$r(t)=\frac{1}{m}\sum_{i=1}^{m}r_{c,i}(t)$$
5. The original signal x(t) can be recovered by removing the added white noise, as shown in (1–10).
  (9) $$x(t)=\sum_{j=1}^{n}{IMF}_{j}(t)+r(t)$$

#### 2.4.3. Denoising Method Based on SVD

Singular Value Decomposition (SVD) is a matrix decomposition technique. According to its decomposition principle, a one-dimensional time series of visible light spectra will be reconstructed into an *m* × *n* matrix *P* after decomposition [31]. The specific representation of matrix *P* is as follows:

(10) $$\begin{array}{l} P={\left[IMF_{1},IMF_{2},\ldots IMF_{m}\right]}^{T}\\ =\left(\begin{array}{ccc} \begin{array}{l} x_{11}\\ x_{21} \end{array} & \begin{array}{l} x_{12}\cdots \\ x_{22}\cdots \end{array} & \begin{array}{l} x_{1n}\\ x_{1(n+1)} \end{array}\\ \vdots & \vdots \ddots & \vdots \\ x_{m1} & x_{m2}\cdots & x_{m(m+n-1)} \end{array}\right) \end{array}$$

In this expression, IMFi represents $\left\{x_{11},x_{12}\cdots ,x_{1n}\right\}$, indicating *N*, the quantity of true IMF components, which describes the measured waveform data. After performing Singular Value Decomposition on matrix P, three matrices can be obtained: *U* is an *m* × *m* matrix, *VT* is an *n* × *n* matrix, and the diagonal matrix *S* is of the size *m* × *n*. The relationship among the three can be expressed with the following formula:

(11) $$P=USV^{T}=\sum_{i=1}^{k}\sigma_{i}u_{i}{v_{i}}^{T}$$

In this expression, *U* ∈ *Rm* × *m* is the left singular matrix, and *VT* ∈ *Rn* × *n* is the right singular matrix. Additionally, the rank of matrix *P* is denoted as *K* [satisfying ≤ min(*m*,*n*)], and typically, *m* is significantly smaller than *n*. The diagonal matrix *S* is in the form *S* = diag (*σ*1, *σ*2, …, *σ*k), where the diagonal elements (*σ*1, *σ*2, …, *σ*k) are the singular values of matrix *P* and the relationship *σ*1 ≥ *σ*2 ≥ … ≥ *σ*k > 0 holds. Furthermore, *u_i_* and *v_i_* represent the *i*-th eigenvectors of the *P^T^P* and *PP^T^* matrices, respectively.

#### 2.4.4. Application of EEMD-SVD Denoising Scheme in Water Quality Detection

When using EEMD for signal denoising, the processing of Intrinsic Mode Functions (IMFs) inevitably leads to the loss of low-frequency useful signals and residual high-frequency noise. Additionally, the determination of the effective rank in the SVD method is easily affected by the trend term of the signal. Considering the non-stationary and stochastic characteristics of visible light spectra, this study proposes a novel denoising method that combines traditional EEMD with SVD. This method aims to effectively remove invalid interferences in the spectra while preserving waveform information as much as possible, building on the foundation of traditional EEMD and SVD. The core functions of the EEMD-SVD denoising model include: (1) significantly reducing the mode mixing effect in Empirical Mode Decomposition (EMD); (2) extracting the trend term of the signal through Ensemble Empirical Mode Decomposition (EEMD) to enhance the adaptivity of singular value selection in Singular Value Decomposition (SVD), thereby effectively addressing the suboptimal denoising issues that may arise from the simple threshold processing of IMFs [32,33]; and (3) effectively suppressing the impulse noise present in visible light spectra.

The specific implementation steps of the EEMD-SVD denoising model are as follows:

(1) Addition of White Noise: Gaussian white noise with a mean of 0 and uniformly distributed is repeatedly added M times (M > 1) to the original signal;

(2) EMD: The visible light spectra with added white noise are decomposed using EMD technology. Through this method, multiple empirical mode components IMF*i* (*I* = 1, 2, …, *n*) are obtained;

(3) Selection of IMF Components: The selection of IMF components is based on the relationship between the correlation coefficient *ρ* and the variance contribution rate *v*. In this process, a similarity metric is introduced, with its applicable range defined as 0 < *λ* < 1. A higher similarity value indicates a stronger degree of similarity. Typically, the similarity threshold is set at 0.95. Only when an IMF component meets this criterion is it considered valid. If these conditions are not met, the component is deemed invalid. Ultimately, the effective IMF components are selected for signal reconstruction. The threshold *λ* satisfies the following conditions:

(12) $$\lambda \leq \frac{\sum_{j=1}^{m}(\rho_{j})+\sum_{j=1}^{m}v_{j}}{2}$$

In the equation, under the condition *m* ≤ *M*, the components from 1 to *m* are extracted in ascending order, corresponding to the indices of the selected valid intrinsic mode components (IIMF);

(4) Construction of the Hankel Matrix: During the construction of the Hankel matrix, it is essential to rely on the optimized IMF matrix. Correctly determining the embedding dimension m and the time delay is crucial for processing the optimized IMF components in phase space reconstruction. The dimension and number of reconstruction vectors are determined by the embedding dimension m, which can affect the number of non-zero singular values obtained through Singular Value Decomposition (SVD). Additionally, the time delay in the time series significantly impacts the correlation between adjacent data points. The appropriate ϵ is selected by analyzing the autocorrelation of the spectral sequence, and the embedding dimension m is determined according to Takens’ theorem: m = (N + τ)/(τ + 1) (where *N* is the number of sampling points). Ultimately, the high-dimensional reconstructed data matrix P is organized into a Hankel matrix;

(5) Selection of SVD Rank: When performing Singular Value Decomposition (SVD), the choice of decomposition rank is a critical factor. Before conducting SVD on the visible light spectrum, determining the decomposition rank of the Hankel matrix is the first step. The selection of this rank primarily considers the number of rows m and columns n of the Hankel matrix, with the goal of maximizing the product of these two values. Research and experimental results show that the parity of the sample size *N* significantly affects the product of m and n, and that m can be derived from the expression m = *N* + 1 − *n*. Once the appropriate SVD rank is identified, the singular values can be extracted using SVD technology;

(6) Determination of Reconstruction Rank in SVD: When performing SVD reconstruction, selecting an appropriate reconstruction rank k is crucial, as it significantly affects the denoising performance. Different ranks can lead to substantial differences in denoising effectiveness. If the reconstruction rank is too low, important signal information may be lost, leading to waveform distortion. Conversely, if the rank is set too high, the noise may not be effectively removed, compromising the denoising effect. To address this, this study employs the method of analyzing the energy distribution of singular values to determine the optimal reconstruction rank. The energy distribution of singular values is examined, and a point k is chosen where the energy difference between adjacent singular values is maximized and the energy curve becomes smooth from that point onward. This indicates that the optimal reconstruction rank for the matrix after SVD should be set to k, effectively distinguishing between signal and noise;

(7) Signal Reconstruction: By reconstructing the matrix data according to the principles of SVD, a one-dimensional time series is ultimately obtained, yielding the denoised signal.

#### 2.4.5. Processing and Analysis of Spectral Images

**Figure 2:**
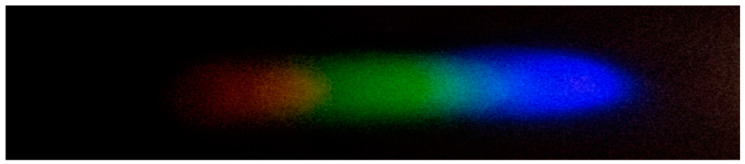
Image affected by noise.

**Figure 3:**
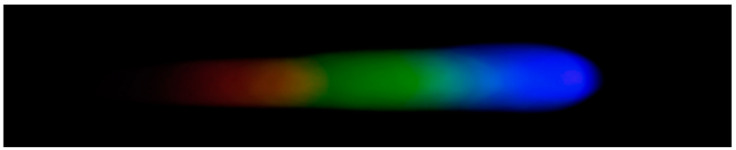
Image obtained after denoising with EEMD-SVD.

After the preparation of the standard solutions, the corresponding color-developing agents are added to each solution in sequence to enable the formation of complexes with hexavalent chromium ions, mercury ions, lead ions, arsenic ions, and copper ions, thereby causing coloration. Through image processing and spectral analysis, the absorption spectra of each ion are obtained to facilitate subsequent analysis and detection [34]. Considering that external lighting may interfere with the experimental results, a dark box is used for the optical path during imaging, with only an LED serving as the light source. It should be noted that there is a certain amount of noise in the image (as shown in Figure 2). To achieve denoising, the EEMD-SVD algorithm is applied to the image to remove noise and generate a new image (as shown in Figure 3). Subsequently, the processed image is cropped to form a 2500 × 700 window. After extracting the meaningful part, the RGB channels are processed. In this operation, quantization is performed using either the HSV model or the grayscale model, ultimately presenting the relationship between pixel intensity and signal intensity in the form of curves. All steps and operations related to image processing and curve drawing are completed on the MATLAB 2017a platform.

The analysis and processing of data form the core content of the research, and this process is based on the Beer–Lambert Law. Additionally, a mathematical relationship (Equation (13)) between the quantization values in the color quantization model and the sample concentration is established.

(13) $$A=\log_{10}\frac{1}{T}=\log_{10}\frac{1}{I_{j}}=ac+b$$

The symbol A represents the absorbance of light, while *T* denotes the transmittance of the various color models. The parameter *c* refers to the concentration of the solution, *I* represents the incoming light quantization data under different color models, and *I*_j_ indicates the quantized measurement values of the standard solution at various concentrations. In the formula, the constants *a* and *b* are fixed parameters. In the grayscale model, *I* typically represents the grayscale reading value of the blank standard solution, while *I*_j_ corresponds to the grayscale values of the standard solutions at different concentrations.

#### 2.4.6. Neural Network Modeling

(1) Convolutional Neural Networks (CNNs)

Convolutional Neural Networks (CNNs) are the most representative neural network structures in the field of image recognition. The local connectivity and parameter sharing employed by CNNs significantly reduce the model’s complexity and the number of parameters, thereby lowering the risk of overfitting. This makes CNNs highly advantageous for processing two-dimensional image data [35]. In the proposed method, CNNs are utilized for image classification and recognition.

(2) Heavy Metal Ion Water Quality Parameter Prediction Model

The best quantification model for the absorption spectra of heavy metal ions is the grayscale model. Therefore, we can establish a CNN prediction model using the grayscale images of the visible absorption spectra. To mitigate the overfitting issue in neural networks, data augmentation is applied to the existing data samples. Without actually increasing the data volume, this approach makes the limited amount of data appear to be larger, ensuring continuous detection and improving the detection accuracy to the hundredth place based on the original sample data. The data augmentation methods used in this study are mainly divided into two categories:

Geometric Transformations: These operations involve flipping, rotating, translating, and cropping images. Such transformations do not change the actual content of the image, but may select a portion of the image or adjust the distribution of pixels.

Color Transformations: These change the content of the image itself, primarily by randomly adding noise (such as Gaussian noise).

Additionally, due to the large number of parameters in the neural network used in this study (134 million parameters shown in Table 1), training such a massive network remains challenging even with data augmentation. Therefore, transfer learning is considered to achieve heavy metal concentration predictions based on this neural network.

**Figure 4:**
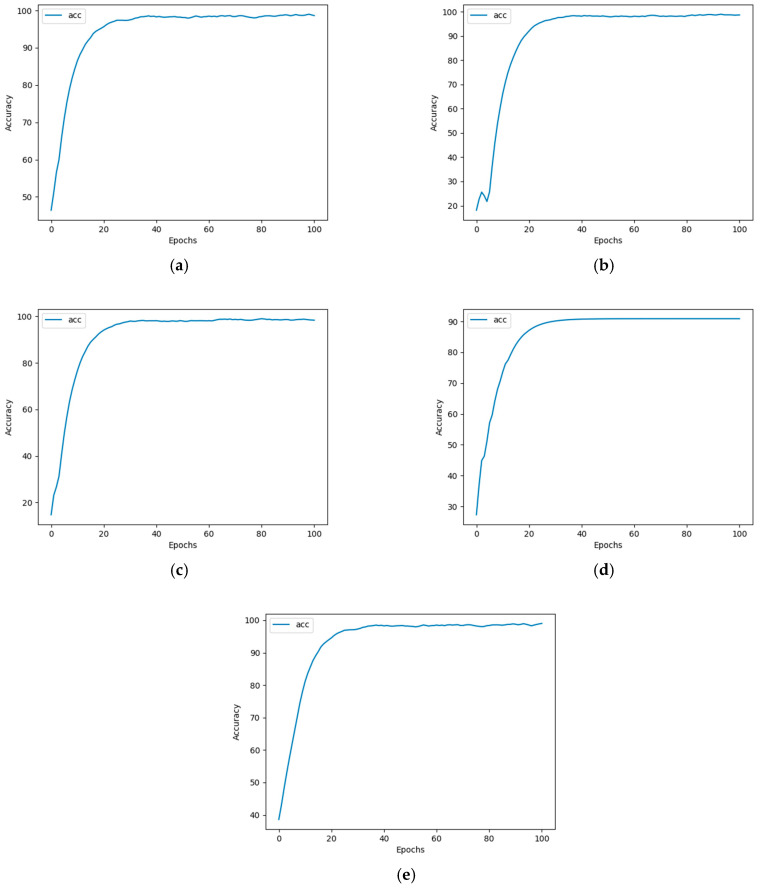
Accuracy of five heavy metal solution samples after 100 epochs of neural network training. (**a**) Cr; (**b**) Hg; (**c**) Pb; (**d**) As; and (**e**) Cu.

During training, as the number of epochs increases, the validation accuracy also gradually improves (see Figure 4). It can be observed that samples of the five heavy metal solutions can converge after 100 epochs of neural network training, with regression accuracy reaching over 97%.

After training, the detection accuracy of the concentrations of the five metal solutions based on the test set data samples is shown in Table 2.

## 3. Design of the Multi-Parameter Water Quality Detection System

**Figure 5:**
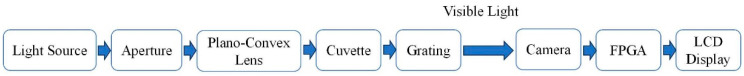
Schematic diagram of the detection system structure.

The water quality detection system established in this study integrates multiple aspects, including optical path structure, hardware, and software systems. The optical path design draws on the principles of visible spectrophotometry, and the specific design details can be seen in Figure 5. Based on the Beer–Lambert Law, the system includes a light source, a plano-convex lens, an aperture, a grating, and a cuvette, utilizing a visible light source to take advantage of the absorption characteristics of lead, copper, mercury, arsenic, and hexavalent chromium ions in visible light. The visible light source for the continuous spectrum in this detection system ranges from 390 nm to 780 nm. A white-light LED powered by three AA batteries (DC 3–5 V) is used. The monochromator disperses composite light into single-wavelength light. Though 100% monochromatic light is not achievable, bandwidth is crucial. Narrower bandwidth enhances analytical sensitivity and ensures a linear relationship between substance concentration and optical response. There are two main types of monochromators. Prism systems, simple in structure but with limited resolution, are unsuitable for miniaturized devices. Grating systems, offering a wide wavelength range, uniform dispersion, and high resolution, are ideal for portable instruments. In this study, the monochromator, combining a diaphragm, plano-convex lens, and holographic diffraction grating, boosts spectral analysis performance.

The diaphragm is a device used to control the intensity and flux of light beams. Gratings are divided into two types: transmission gratings and reflection gratings, the latter including plane reflection gratings and concave reflection gratings. In grating production, it is essential to ensure extremely high precision to create numerous equal and parallel slits, typically forming equidistant grooves on metal or glass. The function of the plano-convex lens is to convert the light filtered by the diaphragm into parallel rays. Gratings can be classified into engraved gratings and holographic gratings based on manufacturing techniques. When composite light illuminates the grating, the combined effects of single-slit and multi-slit diffraction result in the uniform distribution of the spectrum at different wavelengths, with equidistant spectral lines. Holographic technology has significantly increased the groove density of gratings, typically reaching dozens or thousands of lines per millimeter, thereby enhancing the dispersion rate and resolution of the grating. The names, models, and specifications of each component in the monochromator are detailed in Table 3 below.

The software module covers multiple functions, including camera configuration, image data capture, and conversion to grayscale images. Additionally, it implements visible spectrum denoising (based on EEMD-SVD technology), Convolutional Neural Network prediction, AD data acquisition, LCD display driving, and user interface presentation. The development primarily utilizes two parts: Verilog language programming and the Vivado 19.1 platform. The hardware system consists of a camera, an FPGA control chip, and an LCD display. The FPGA used is the Xilinx Zynq UltraScale + MPSoC XCZU19EG, a powerful FPGA development board that combines ARM Cortex-A53 and Cortex-R5 processing cores with programmable logic (FPGA) resources, suitable for the design and development of complex systems. The challenge of porting the Convolutional Neural Network to the ZYNQ platform has been overcome. After model quantization, compression, and acceleration design, the data captured by the camera is cached to the DDR memory on the PS side via VDMA using the AXI protocol and then passed to the accelerator to generate the classification results, successfully displayed in the form of a DVP video stream on the LCD screen. This realizes the rapid detection of heavy metal ion concentrations in water based on the ZYNQ spectral detection system.

## 4. Case Study: Copper Ions in Water

### 4.1. Experimental Instruments and Materials

The experimental materials were as follows: copper ion standard solution and coloring reagent. The experimental instrumentswere as follows: high-precision electronic analytical balance (accuracy of 0.00001 g) provided by Licheng Technology, a single-channel pipette (accuracy ± 1.5%) with a specification of 100–1000 µL, provided by JOANLAB, beakers, and volumetric flasks.

Accurately pipette 100 mL of the copper ion standard solution (Cu^2+^ = 1000 mg/L). According to the laboratory method, use the pipette to further dilute the standard solution to prepare ten sets of test solutions with concentrations of 0.1 mg/L, 0.2 mg/L, 0.3 mg/L, 0.4 mg/L, 0.5 mg/L, 0.6 mg/L, 0.7 mg/L, 0.8 mg/L, 0.9 mg/L, and 1.0 mg/L. The preparation steps for the coloring reagent are as follows: In a beaker, take 0.4 g of dipyridyl oxalate, add 500 mL of 1:1 ethanol solution, heat to 60–70 °C and stir until completely dissolved, then cool to room temperature. Next, dissolve 40 g of ammonium citrate and 28 g of ammonium chloride in about 300 mL of water. After mixing these two solutions, add 20 mL of ammonia solution and finally transfer to a 1000 mL volumetric flask and make up to the mark.

### 4.2. Spectral Image Analysis of Copper Solution

**Figure 6:**
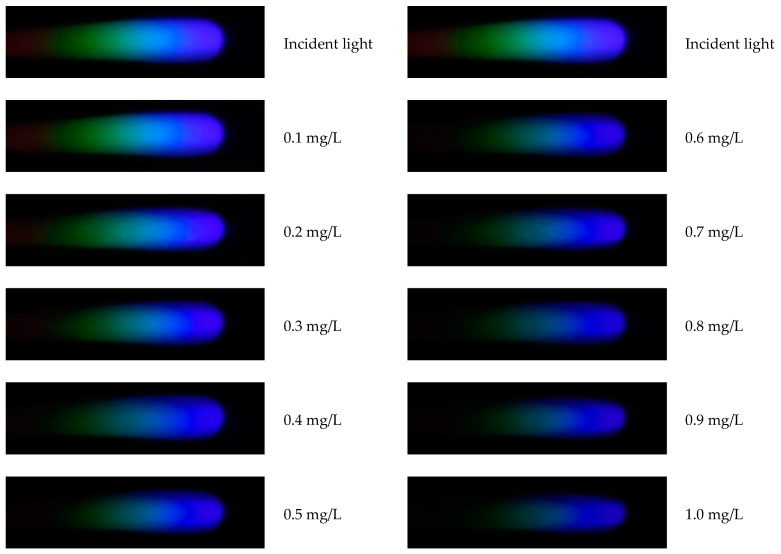
Spectra of copper ion solutions at various concentrations.

**Figure 7:**
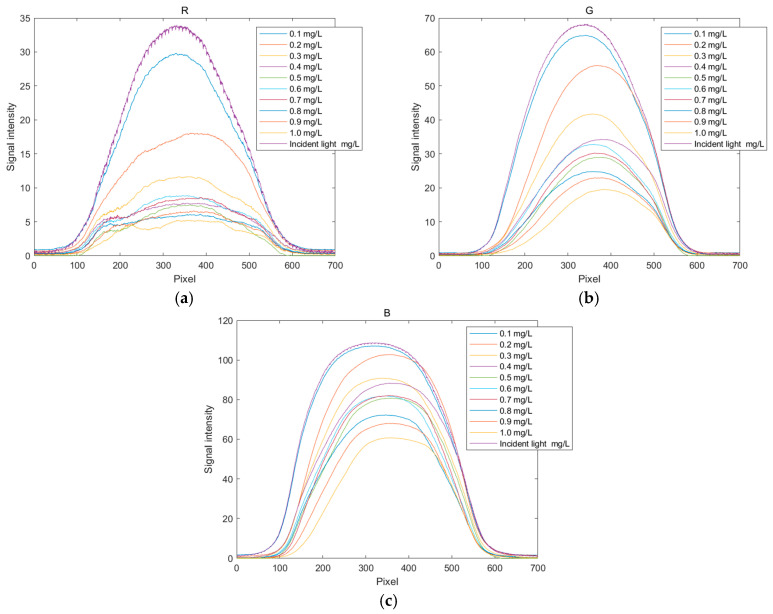
Distribution curves of RGB values for copper ion solutions. (**a**) Distribution curve of R values; (**b**) distribution curve of G values; and (**c**) distribution curve of B values.

After processing with MATLAB, the relationship curves between pixel values and signal intensity can be drawn for images of copper solutions at various concentrations. The spectral diagrams of copper solutions at different concentrations are shown in Figure 6, and the related R, G, and B spectral curves are shown in Figure 7. The copper solution shows a cyan–blue color after coloring, which absorbs orange light according to the complementary color theory. It can be clearly seen from Figure 6 that the R channel value curve undergoes significant changes, indicating that the copper solution has a stronger absorption capacity for red light, resulting in a lower energy peak in the red value distribution curve. As the concentration of the copper solution increases, the red value distribution curve will further decrease. From Figure 7, it can also be seen that the G channel changes significantly, with higher absorption of green light by the copper solution. Compared to other channels, the B channel value curve changes less. Therefore, in the R, G, and B channels, the positions with significant changes in pixel values were selected as 350, 350, and 300, respectively, to establish the corresponding standard curves based on these quantization values.

**Figure 8:**
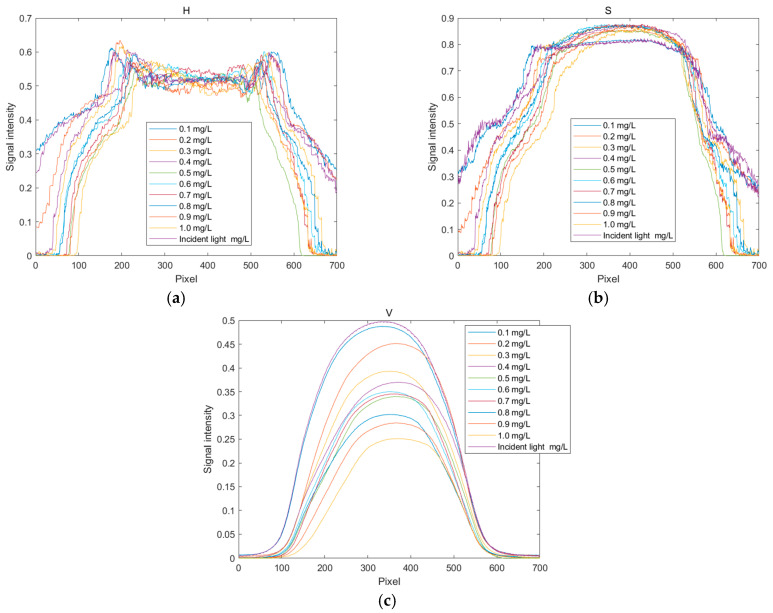
Distribution curves of HSV values for copper ion solutions. (**a**) Distribution curve of H values; (**b**) distribution curve of S values; (**c**) and distribution curve of V values.

The distribution curves of the H, S, and V values for copper solutions at different concentrations are shown in Figure 8. Since these solutions appear cyan–blue, their hue (H) value hardly changes significantly with an increasing concentration, as seen in Figure 8a. Figure 8b,c illustrate the changes in saturation (S) values with the concentration of the standard solution. The observations indicate that the changes in saturation are more pronounced in the red region, while they are less noticeable in other color regions. Within the range from yellow–green to red, the brightness decreases gradually with an increasing concentration, indicating enhanced light absorption in this region. Therefore, when establishing the standard curves for the H, S, and V indicator values, the quantization values at pixel positions 200, 350, and 350 are selected in sequence.

**Figure 9:**
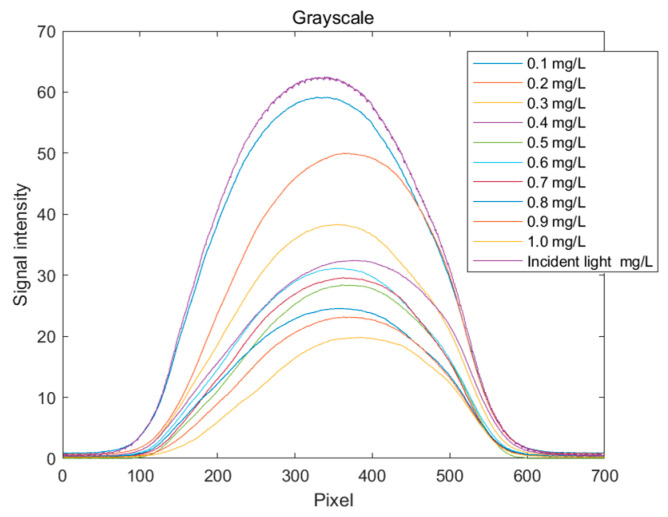
Distribution curve of grayscale values for copper ion solutions.

The grayscale spectral curves for copper solutions at different concentrations are shown in Figure 9. It can be clearly observed that the grayscale values decrease with an increasing solution concentration. This indicates that more light is absorbed, resulting in less reflected or transmitted light, which in turn reduces the overall image brightness and causes the standard curve of grayscale values to decline. When establishing the standard curve for the grayscale value indicator, the quantization value at pixel position 350, where the change is more pronounced, is selected.

**Figure 10:**
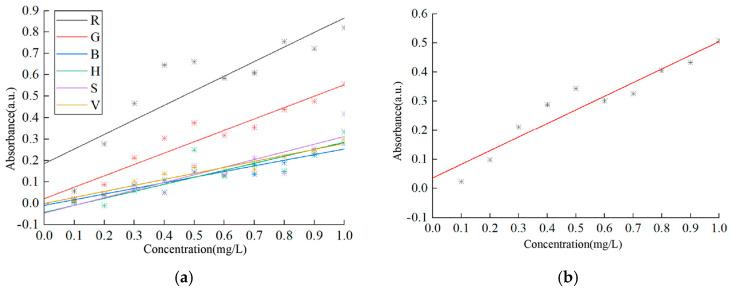
Calibration standard curves of copper solution. (**a**) Standard curve of R, G, B, H, S, and V parameters; (**b**) standard curve of grayscale value parameter.

Therefore, the selected pixel values of R, G, B, H, S, V, and their grayscale values are applied to Equation (13) to calculate the absorbance of copper solutions at different concentrations. After linear regression analysis, the generated standard curves are shown in Figure 10, which indicates that the standard curve based on grayscale values exhibits the highest sensitivity. For detailed data on the intercepts, slopes, and correlation coefficients of different standard curves, please refer to Table 4, where the correlation coefficients of the G value, B value, V value, and grayscale value all exceed 0.9, and the correlation between the grayscale value and copper solution concentration is the most significant. It can be inferred that the model based on the grayscale values is the optimal choice.

### 4.3. Effectiveness Analysis of the EEMD-SVD Spectral Denoising Method

#### 4.3.1. Validation of the Rationality of the EEMD-SVD Spectral Denoising Method

To verify the rationality and practicality of the proposed EEMD-SVD spectral denoising method, the spectra of copper ion (0.3 g/mL), arsenic ion (0.05 g/mL), lead ion (0.03 g/mL), mercury ion (0.0003 g/mL), and chromium ion (0.05 g/mL) solutions were analyzed for denoising. The process involved adding Gaussian noise to the original images and then applying the EEMD-SVD denoising method to the noisy spectra. The results confirmed the superiority of the EEMD-SVD method. Additionally, water sample detection comparative tests for the four heavy metal ions (As, Cr(VI), Pb, Hg) were conducted using ten sets of test standard solutions, following the same method as that used for copper ion detection.

(1) Analysis of the Denoising Effect of Copper Ion Solution Spectra

**Figure 11:**
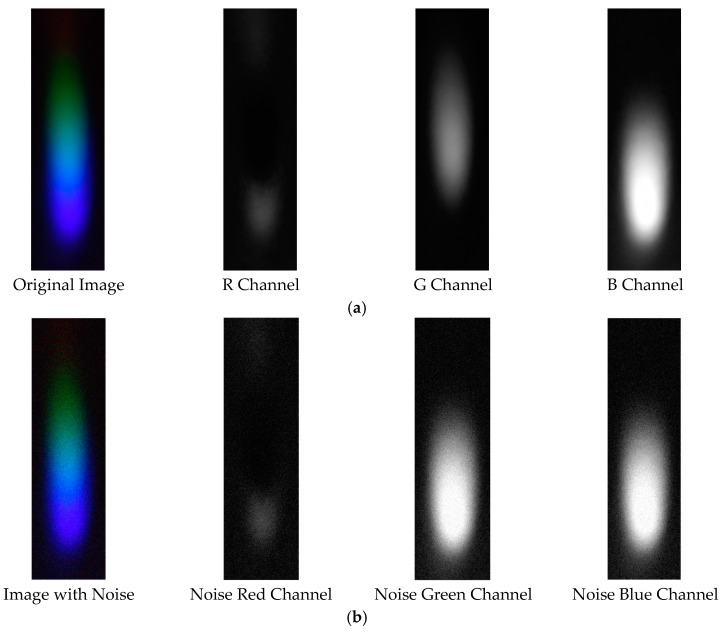
Visible light of 0.3 copper solution. (**a**) Without added Gaussian noise; (**b**) with added Gaussian noise.

The visible light of the 0.3 g/mL copper ion solution without and with added Gaussian noise is shown in Figure 11.

**Figure 12:**
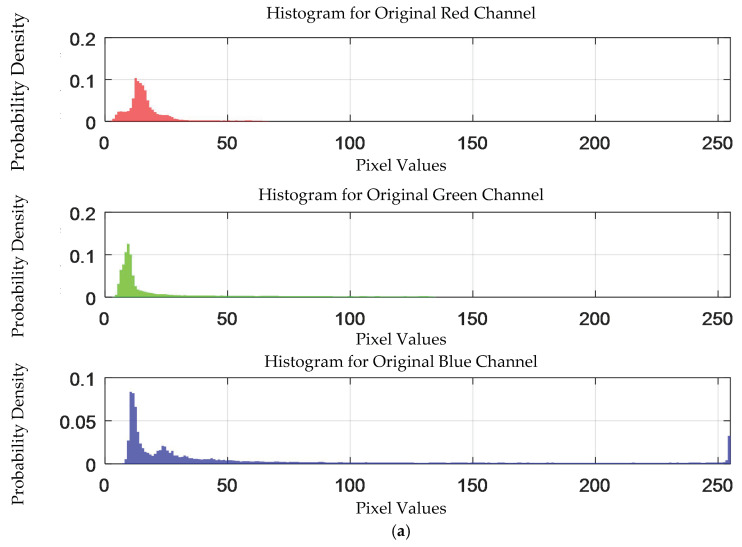

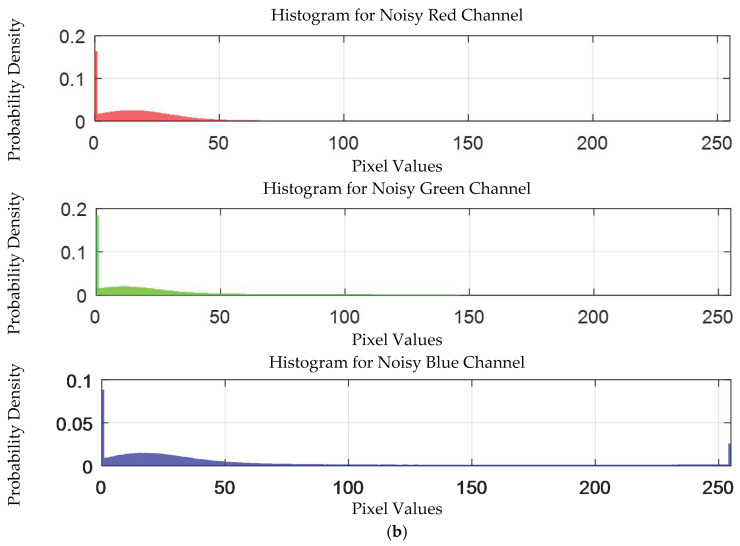
The probability density distribution of RGB values of 0.3 g/mL copper ion solution without and with added Gaussian noise. (**a**) Without added Gaussian noise; (**b**) with added Gaussian noise.

The probability density distribution of the RGB values of the 0.3 g/mL copper ion solution without and with added Gaussian noise is shown in Figure 12.

**Figure 13:**
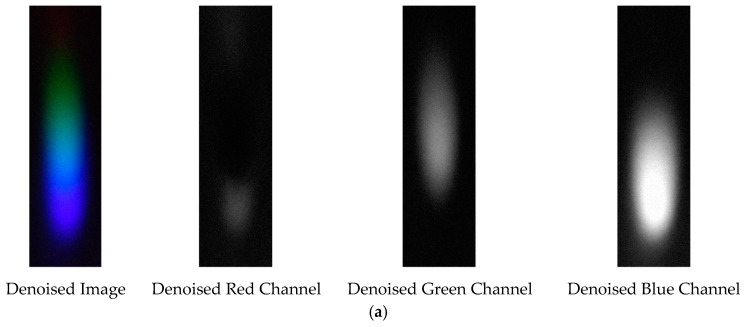

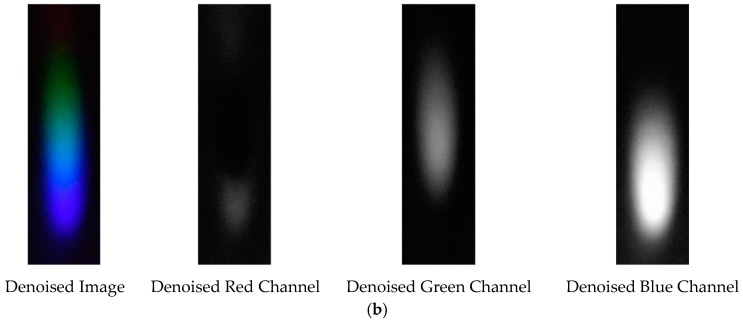
Visible light of 0.3 g/mL copper ion solution after denoising. (**a**) EEMD denoising; (**b**) EEMD-SVD denoising.

The denoising effects of EEMD and EEMD-SVD on the visible light spectrum of the 0.3 g/mL copper ion solution are compared. The results show that EEMD-SVD outperforms EEMD in noise reduction, as seen in Figure 13.

**Figure 14:**
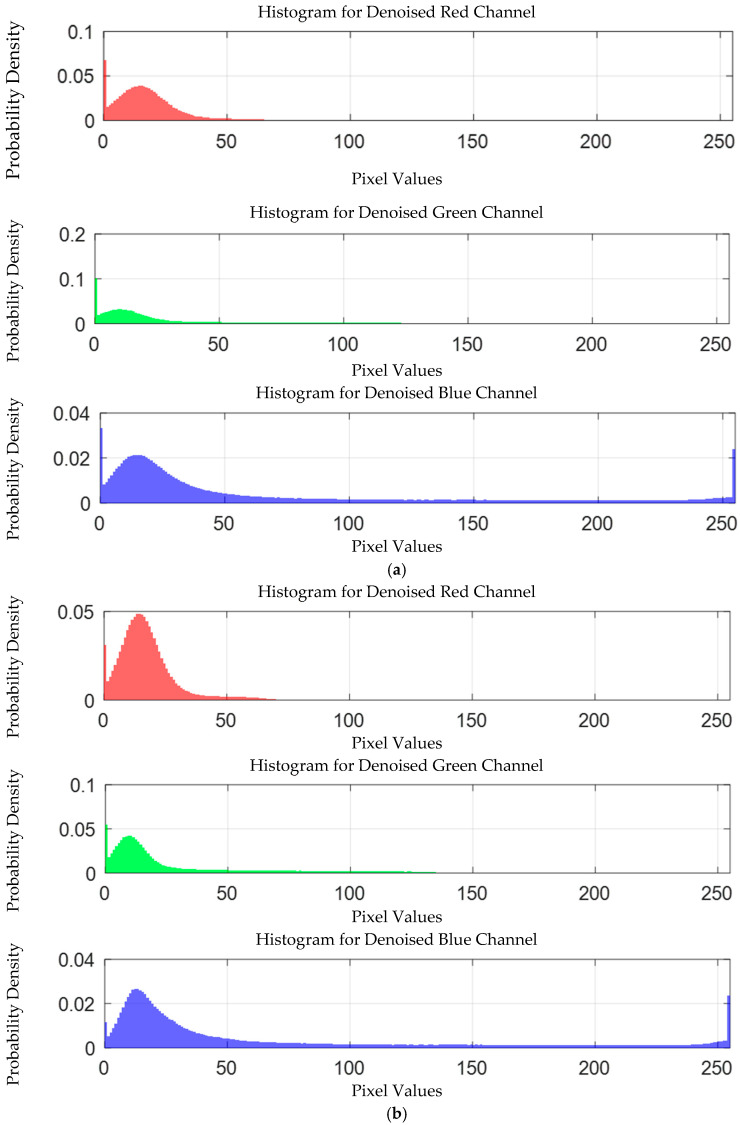
Probability density distribution of RGB values of 0.3 g/mL copper ion solution after denoising. (**a**) EEMD denoising; (**b**) EEMD-SVD denoising.

The probability density distribution of the RGB values of the 0.3 g/mL copper ion solution after EEMD and EEMD-SVD denoising is shown in Figure 14. Compared with the original distribution, EEMD-SVD shows better denoising performance than EEMD.

(2) Analysis of the Denoising Effect of Arsenic Ion Solution Spectra

**Figure 15:**
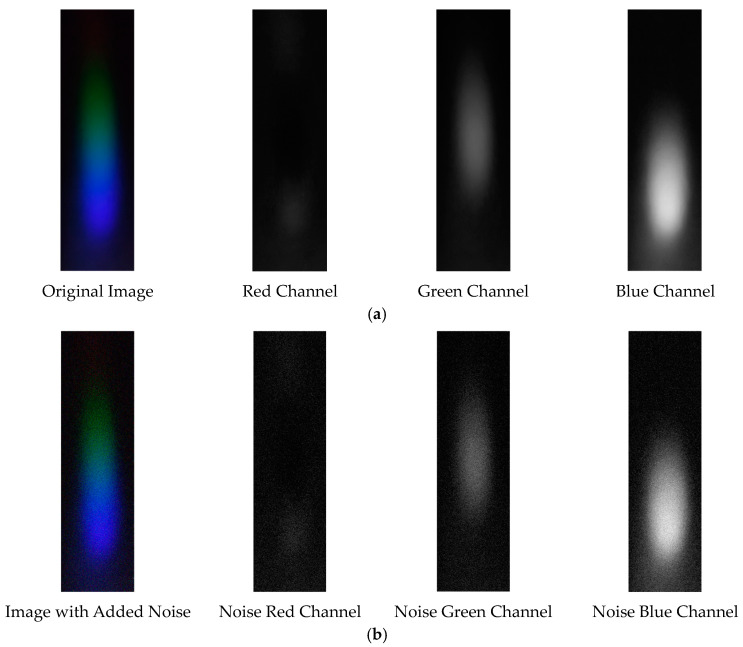
Visible light of 0.05 g/mL arsenic ion solution. (**a**) Without added Gaussian noise; (**b**) with added Gaussian noise.

The visible light of the 0.05 g/mL arsenic ion solution without and with added Gaussian noise is shown in Figure 15.

**Figure 16:**
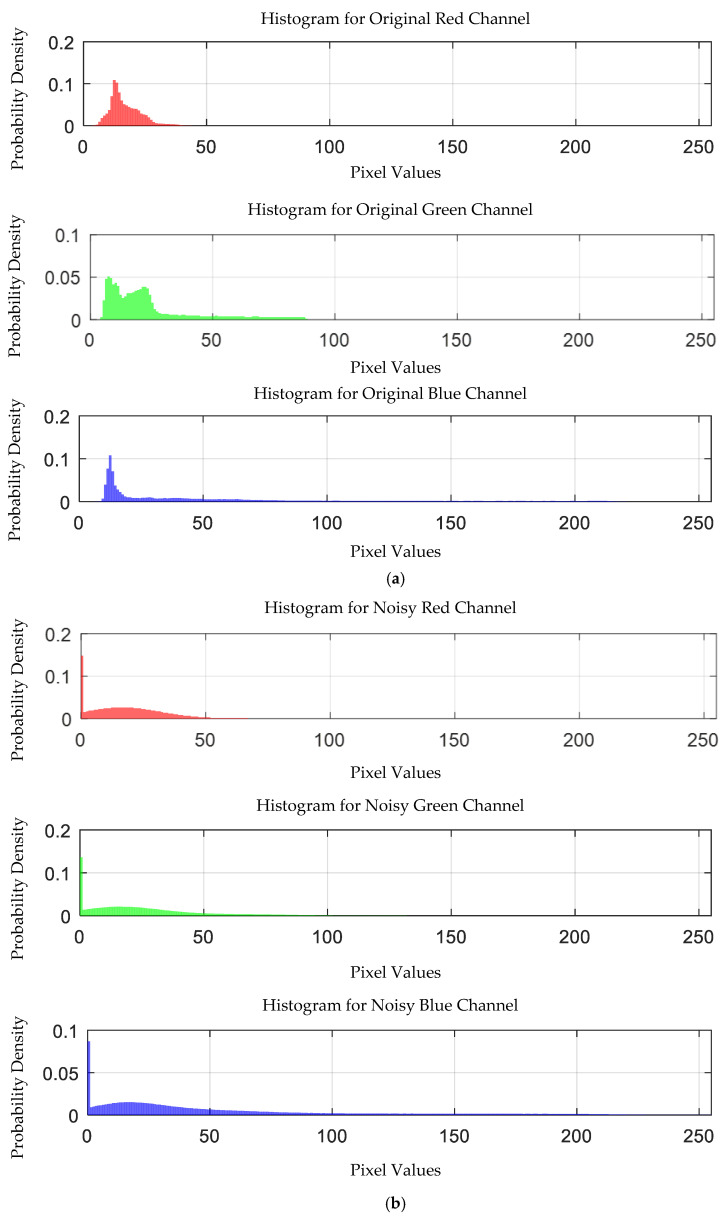
Probability density distribution of RGB values of 0.05 g/mL arsenic ion solution without and with added Gaussian noise. (**a**) Without added Gaussian noise; (**b**) with added Gaussian noise.

The probability density distribution of the RGB values of the 0.05 g/mL arsenic ion solution without and with added Gaussian noise is shown in Figure 16.

**Figure 17:**
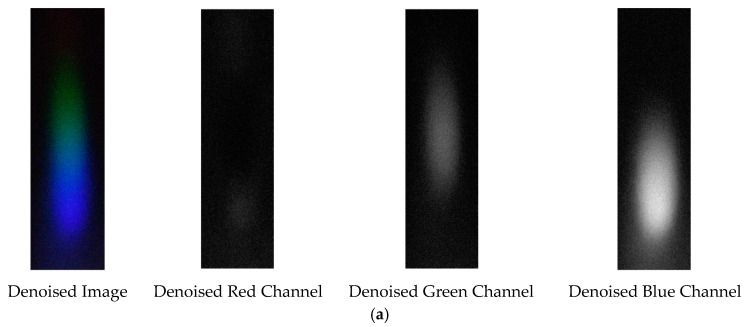

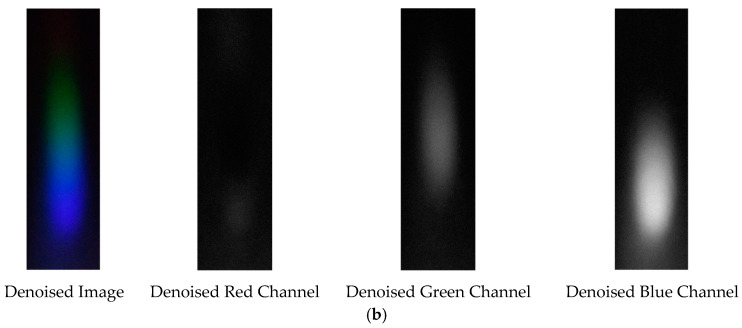
Visible light of 0.05 g/mL arsenic ion solution after denoising. (**a**) EEMD denoising; (**b**) EEMD-SVD denoising.

The denoising effects of EEMD and EEMD-SVD on the visible light spectrum of the 0.05 g/mL arsenic ion solution are compared. The results show that EEMD-SVD outperforms EEMD in noise reduction, as seen in Figure 17.

**Figure 18:**
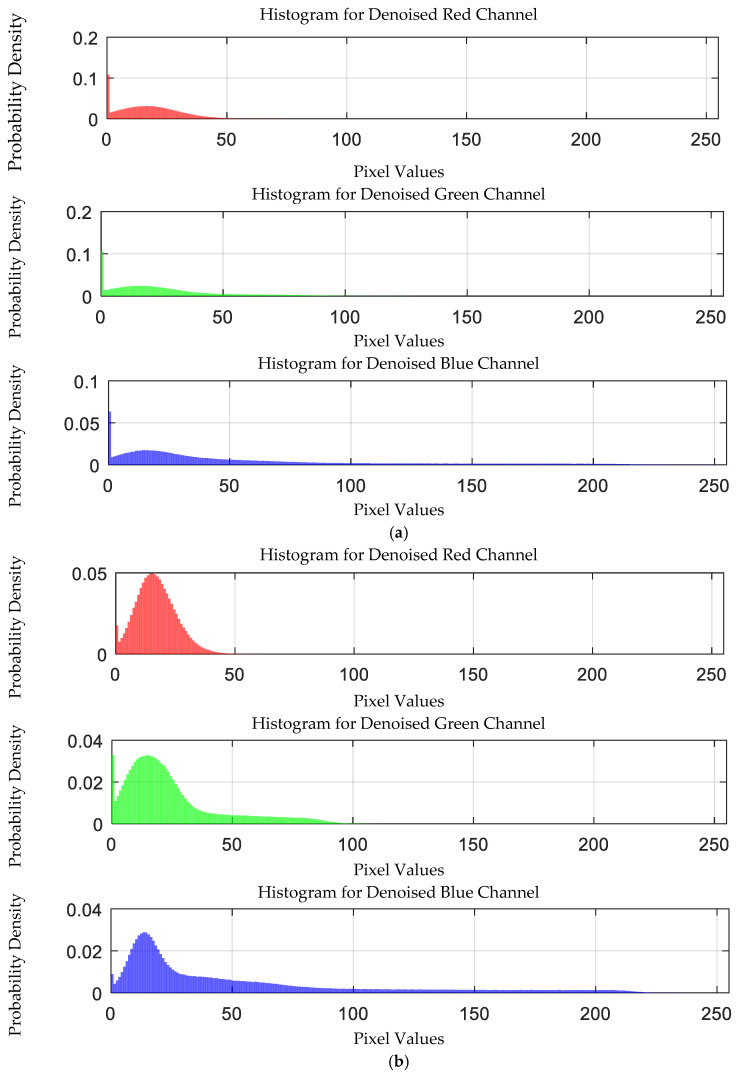
Probability density distribution of RGB values of 0.05 g/mL arsenic ion solution after denoising. (**a**) EEMD denoising; (**b**) EEMD-SVD denoising.

The probability density distribution of the RGB values of the 0.05 g/mL arsenic ion solution after EEMD and EEMD-SVD denoising is shown in Figure 18. Compared with the original distribution, EEMD-SVD shows better denoising performance than EEMD.

(3) Analysis of the Denoising Effect of Lead Ion Solution Spectra

**Figure 19:**
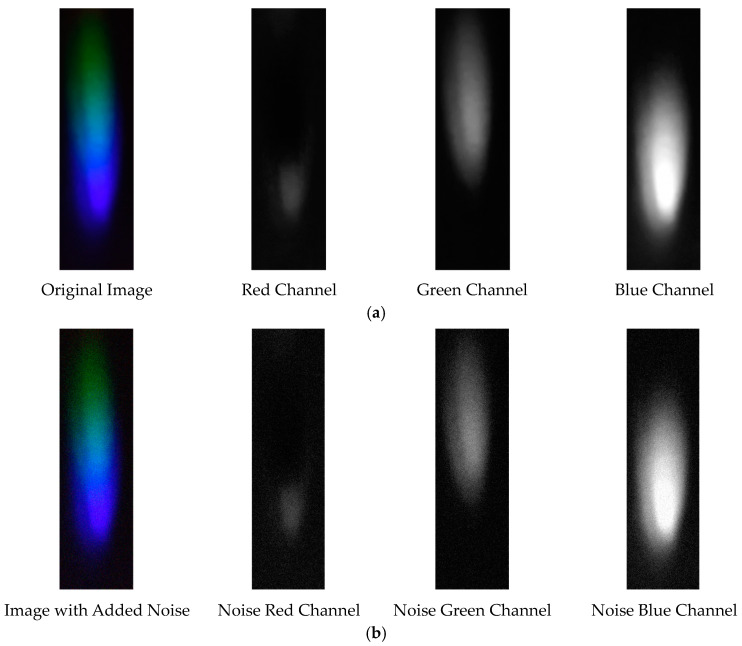
Visible light of 0.03 g/mL lead ion solution. (**a**) Without added Gaussian noise; (**b**) with added Gaussian noise.

The visible light of the 0.03 g/mL lead ion solution without and with added Gaussian noise is shown in Figure 19.

**Figure 20:**
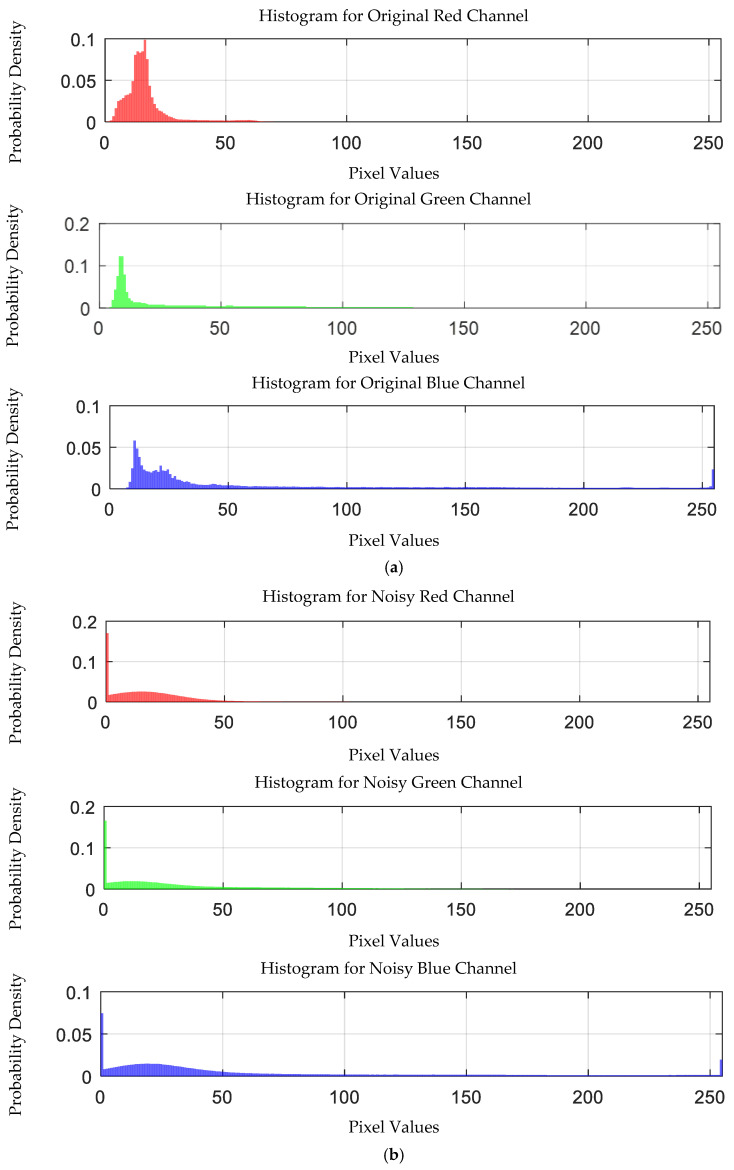
Probability density distribution of RGB values of 0.03 g/mL lead ion solution without and with added Gaussian noise. (**a**) Without added Gaussian noise; (**b**) with added Gaussian noise.

The probability density distribution of the RGB values of the 0.03 g/mL lead ion solution without and with added Gaussian noise is shown in Figure 20.

**Figure 21:**
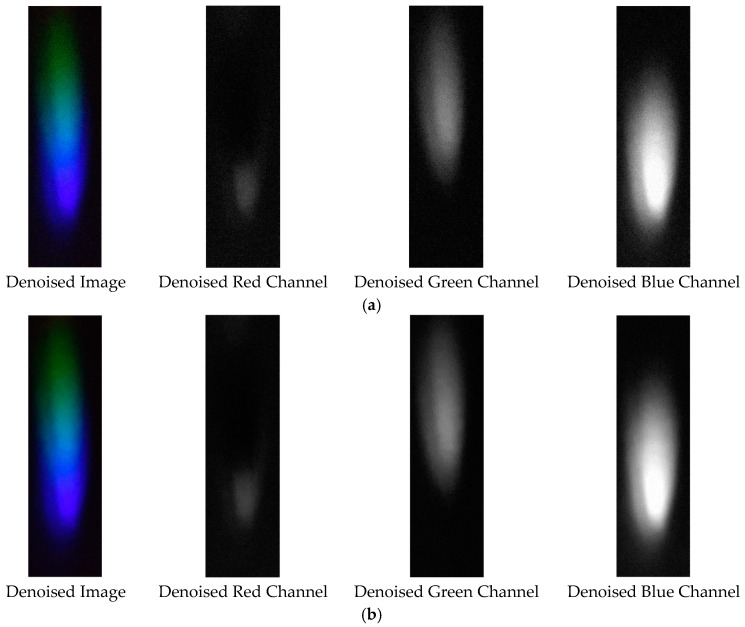
Visible light of 0.03 g/mL lead ion solution after denoising. (**a**) EEMD denoising; (**b**) EEMD-SVD denoising.

The denoising effects of EEMD and EEMD-SVD on the visible light spectrum of the 0.03 g/mL lead ion solution are compared. As shown in Figure 21, EEMD-SVD outperforms EEMD in noise reduction.

**Figure 22:**
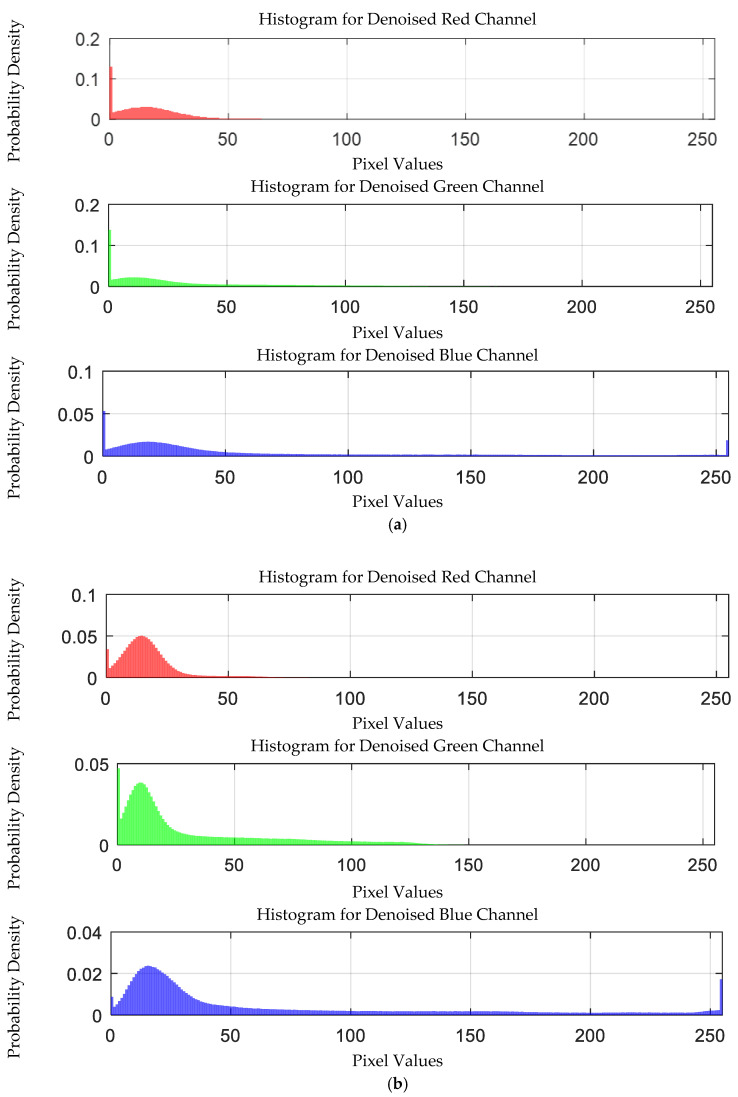
Probability density distribution of RGB values of 0.03 g/mL lead ion solution after denoising. (**a**) EEMD denoising; (**b**) EEMD-SVD denoising.

The probability density distribution of the RGB values of the 0.03 g/mL lead ion solution after EEMD and EEMD-SVD denoising is shown in Figure 22. Compared with the original distribution, EEMD-SVD shows better denoising performance than EEMD.

(4) Analysis of the Denoising Effect of Mercury Ion Solution Spectra

**Figure 23:**
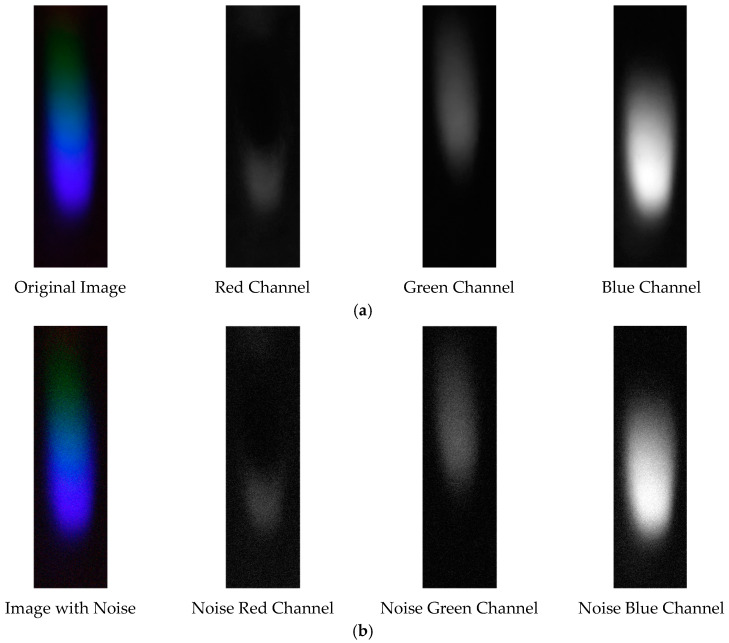
Visible light of 0.0003 g/mL mercury ion solution. (**a**) Without added Gaussian noise; (**b**) with added Gaussian noise.

The visible light of the 0.0003 g/mL mercury ion solution without and with added Gaussian noise is shown in Figure 23.

**Figure 24:**
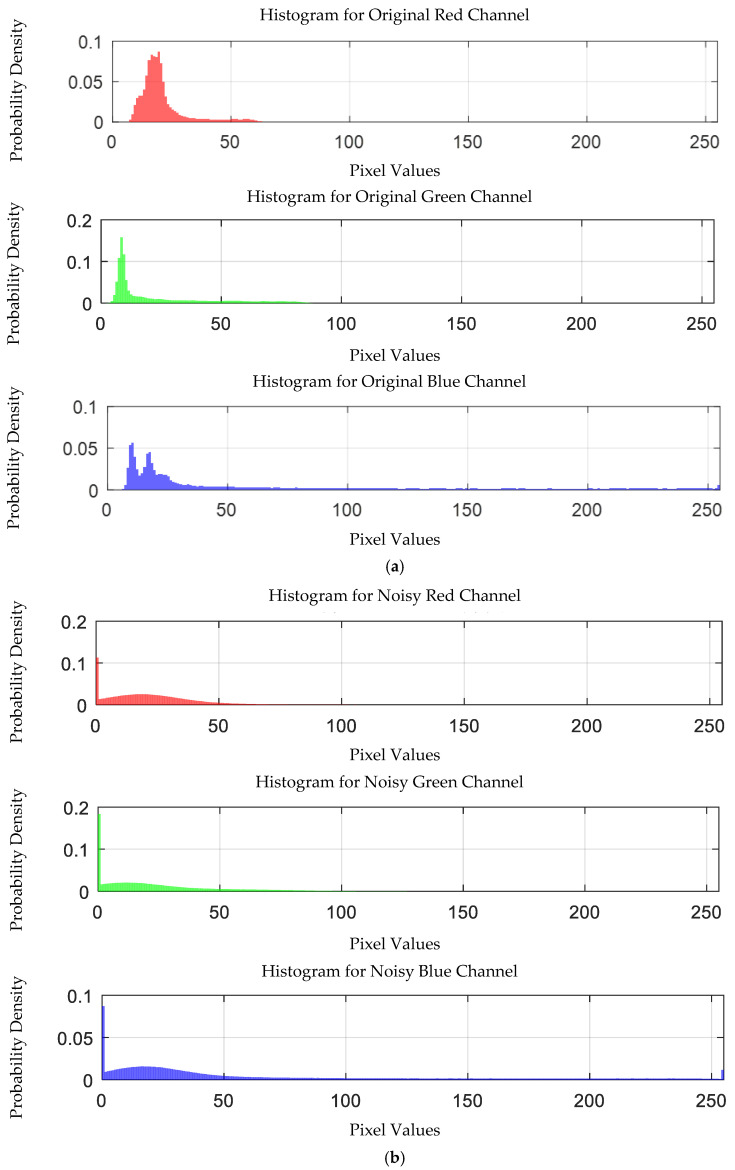
Probability density distribution of RGB values of 0.0003 g/mL mercury ion solution without and with added Gaussian noise. (**a**) Without added Gaussian noise; (**b**) with added Gaussian noise.

The probability density distribution of the RGB values of the 0.0003 g/mL mercury ion solution without and with added Gaussian noise is shown in Figure 24.

**Figure 25:**
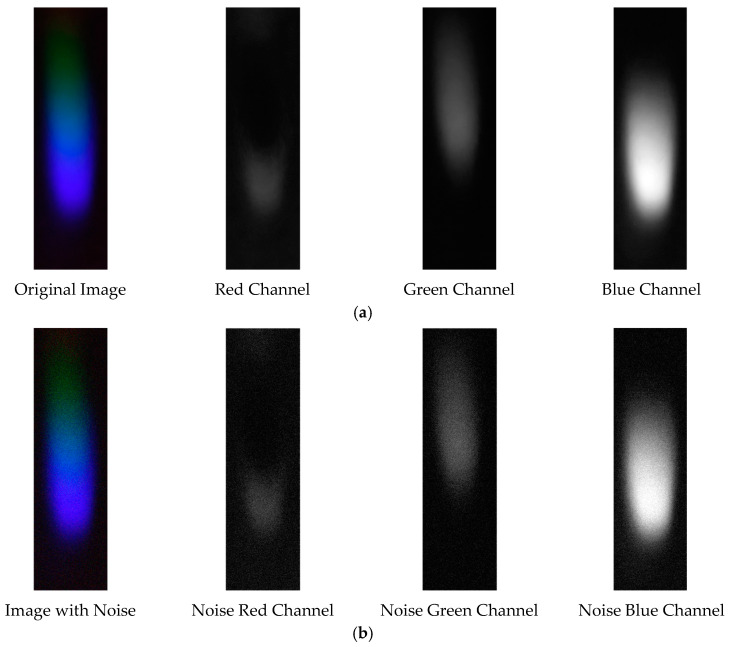
Visible light of 0.0003 g/mL mercury ion solution after denoising. (**a**) EEMD denoising; (**b**) EEMD-SVD denoising.

The denoising effects of EEMD and EEMD-SVD on the visible light spectrum of the 0.0003 g/mL mercury ion solution are compared. As shown in Figure 25, EEMD-SVD outperforms EEMD in noise reduction.

**Figure 26:**
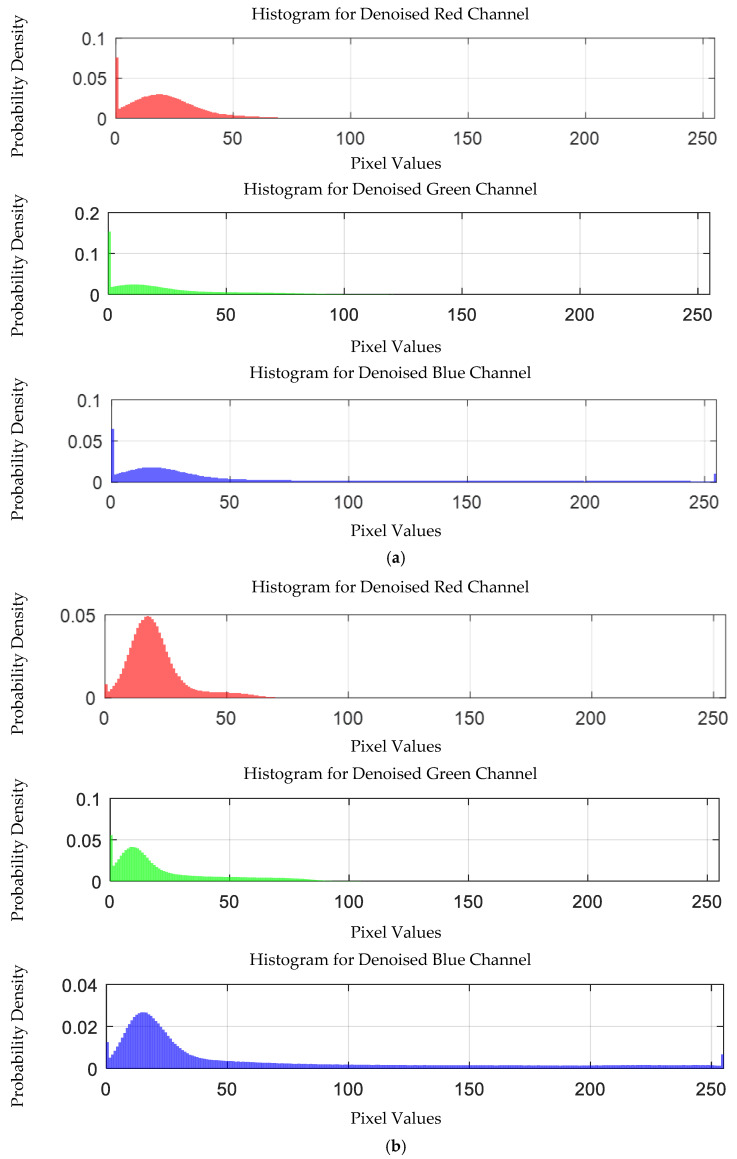
Probability density distribution of RGB values of 0.0003 g/mL mercury ion solution after denoising. (**a**) EEMD denoising; (**b**) EEMD-SVD denoising.

The probability density distribution of the RGB values of the 0.0003 g/mL mercury ion solution after EEMD and EEMD-SVD denoising is shown in Figure 26. Compared with the original distribution, EEMD-SVD shows better denoising performance than EEMD.

(5) Analysis of the Denoising Effect of Chromium Ion Solution Spectra

**Figure 27:**
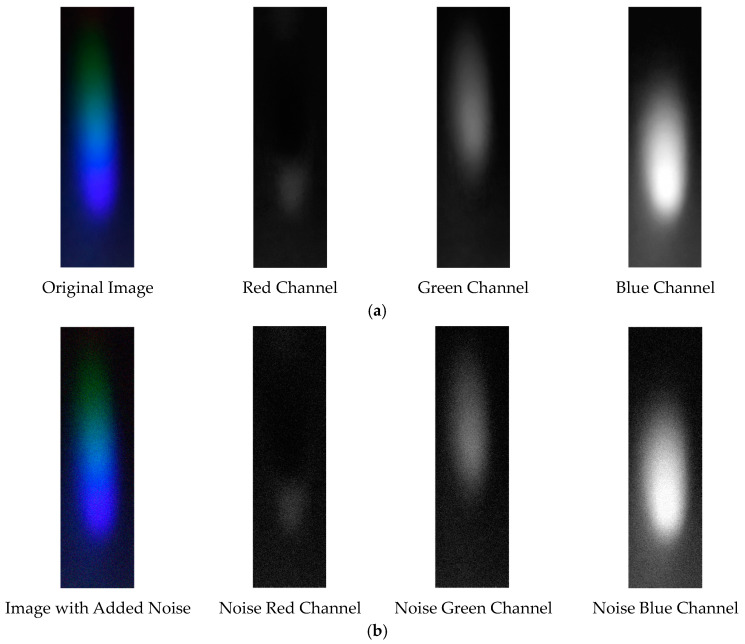
Visible light of 0.05 g/mL chromium ion solution. (**a**) Without added Gaussian noise; (**b**) with added Gaussian noise.

The visible light of the 0.05 g/mL chromium ion solution without and with added Gaussian noise is shown in Figure 27.

**Figure 28:**
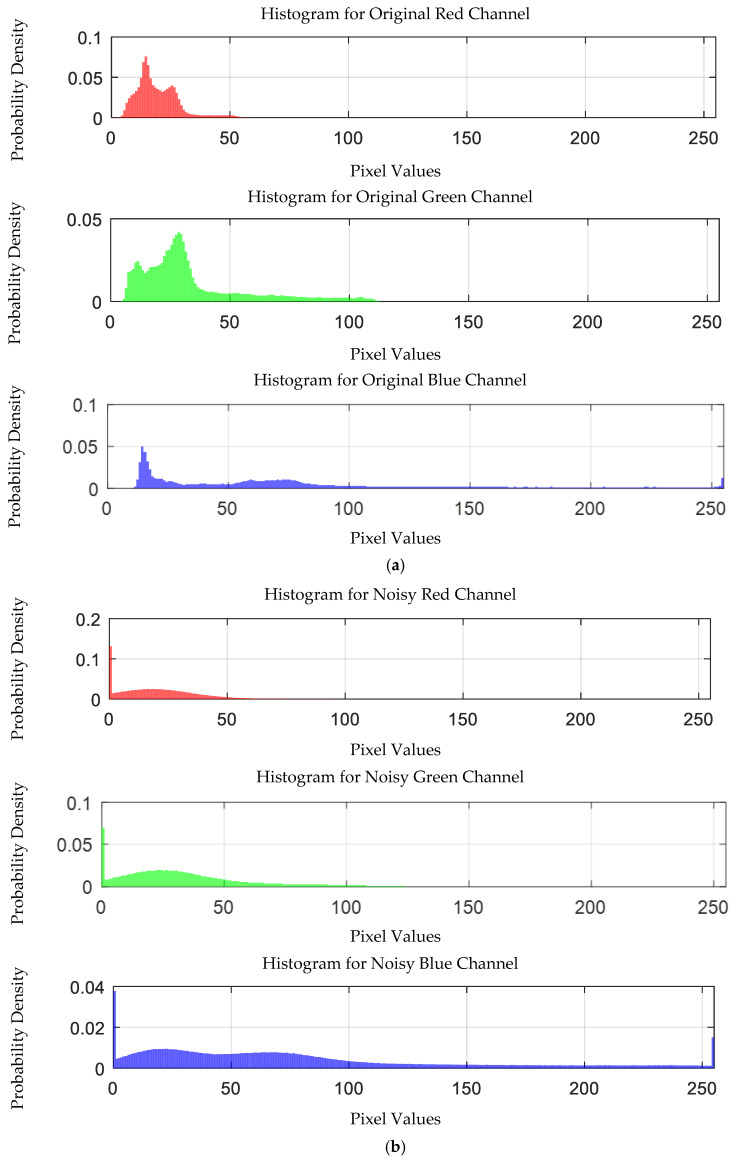
Probability density distribution of RGB values of 0.05 g/mL chromium ion solution without and with added Gaussian noise. (**a**) Without added Gaussian noise; (**b**) with added Gaussian noise.

The probability density distribution of the RGB values of the 0.05 g/mL chromium ion solution without and with added Gaussian noise is shown in Figure 28.

**Figure 29:**
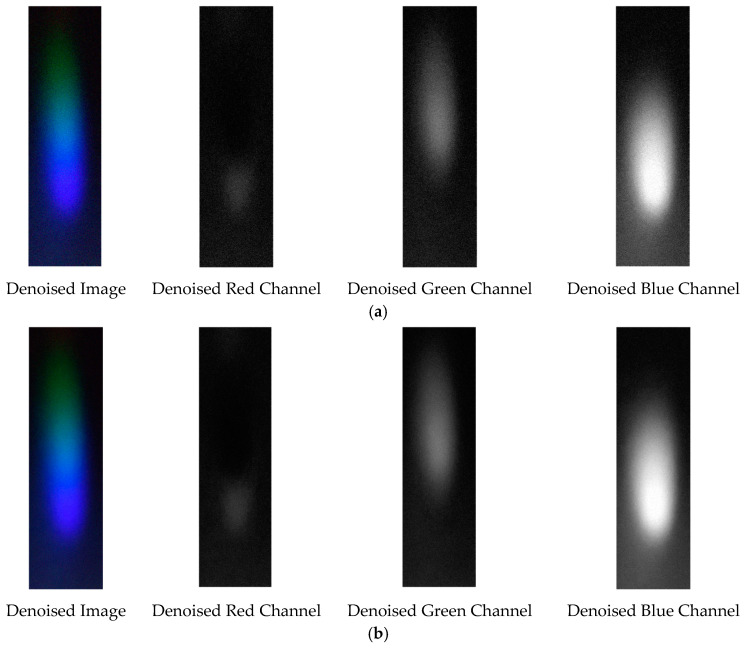
Visible light of 0.05 g/mL chromium ion solution after denoising. (**a**) EEMD denoising; (**b**) EEMD-SVD denoising.

The denoising effects of EEMD and EEMD-SVD on the visible light spectrum of the 0.05 g/mL chromium ion solution are compared. As shown in Figure 29, EEMD-SVD outperforms EEMD in noise reduction.

**Figure 30:**
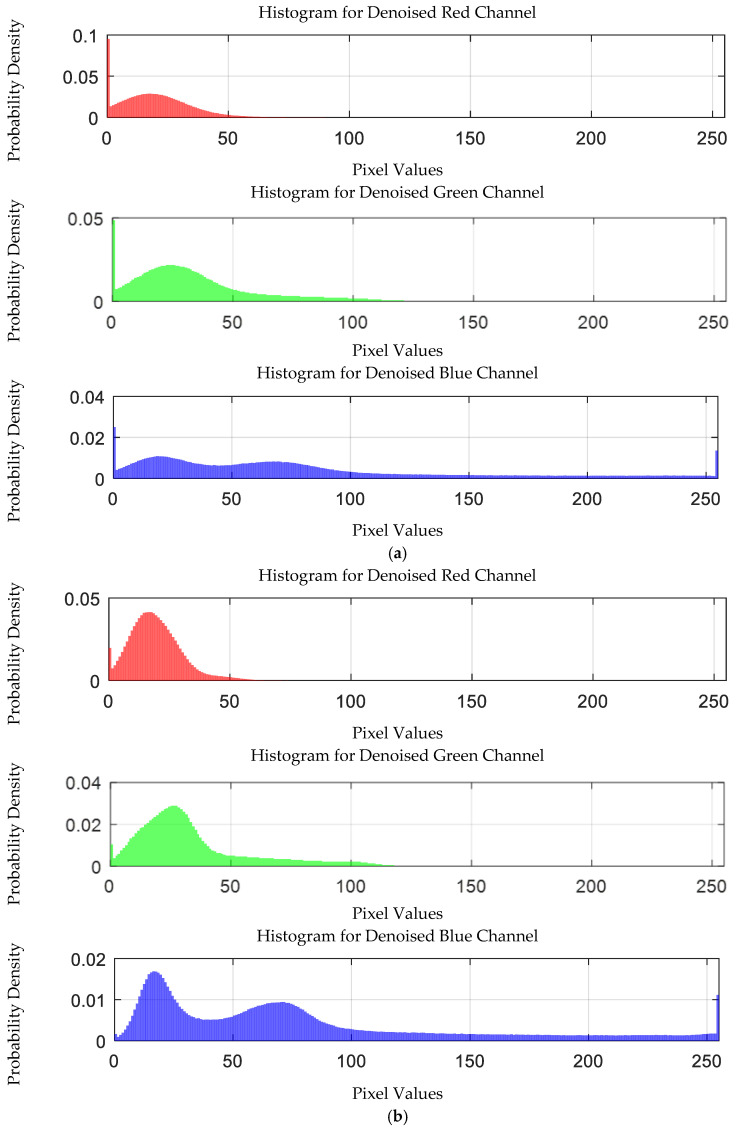
Probability density distribution of RGB values of 0.05 g/mL chromium ion solution after denoising. (**a**) EEMD denoising; (**b**) EEMD-SVD denoising.

The probability density distribution of the RGB values of the 0.05 g/mL chromium ion solution after EEMD and EEMD-SVD denoising is shown in Figure 30. Compared with the original distribution, EEMD-SVD shows better denoising performance than EEMD.

#### 4.3.2. Field Water Sampling and Detection Test Comparison Analysis

**Figure 31:**
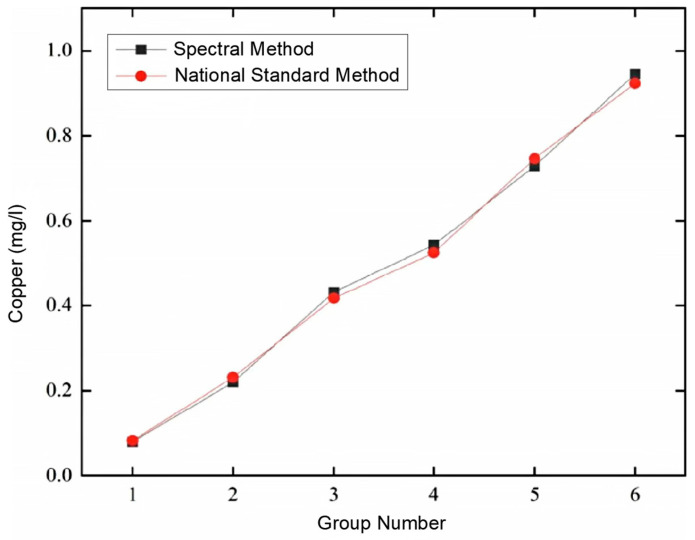
Comparison curve of average copper (Cu) concentration detected using national standard method and spectroscopic method.

Using the prepared 10 sets of test standard solutions, and employing the ZYNQ-based spectral water quality analysis detection system, combined with the national standard analysis method, comparative tests were conducted on copper (Cu) ion solutions. Initially, the preliminary stage of the test involved detecting the concentration of copper (Cu) in heavy metal ion water samples using the Chinese national standard method. Six water samples of copper (Cu) ions were randomly selected from the test set of the current sample library, and each was measured six times. Subsequently, the ZYNQ-based spectral water quality analysis detection system was used to detect the concentration of copper ions in the same water solution, and the test results of the copper ion concentration were compared. The comparative test results are detailed in Table 3. Additionally, the line chart of the average concentration of copper (Cu) metal detected by the national standard analysis method and the ZYNQ-based spectral water quality analysis detection system is shown in Figure 31. Finally, by systematically comparing the obtained data, the effectiveness and accuracy of the two detection methods were evaluated.

As shown in Figure 31, there is good consistency between the national standard detection method and the ZYNQ-based spectral water quality analysis detection system in the determination of the copper (Cu) concentration. From Table 5, it can be seen that the relative error range of the detected copper (Cu) values is between −8.7% and 7.32%, all less than 10%, and the repeatability is less than 4.06%, below the standard requirement of 10%. These results indicate that the detection system meets the technical indicators of the Chinese national environmental protection standard “HJ/T 354-2007 Technical Specification for Acceptance of Water Pollution Source Online Monitoring System”. Moreover, the system has the advantage of rapid detection, fully meeting the needs of water quality detection. It should be noted that the repeatability in Table 5 refers to the degree of consistency between the independent test results obtained via the system when measuring the same water quality parameter in a sample multiple times under the same precision conditions. Repeatability is described using the relative standard deviation (RSD), as shown in Equation (14):

(14) $$RSD=\frac{1}{\bar{C}}\sqrt{\frac{1}{n}\sum_{1}^{n}{\left(C_{i}-\bar{C}\right)}^{2}}$$

In the formula, *Ci* represents the *i*-th measurement result of the same sample, where *i* = 1, 2, …, *n*. $\bar{C}$ is the arithmetic mean of the *n* measurement results. *n*, typically between 5 and 7, was set to 5 in this study.

## 5. Conclusions

This study focused on copper (Cu) ions as an indicator of heavy metal water pollution, aiming to achieve the “rapid and accurate detection of heavy metal ion concentrations using a portable water quality detection system based on spectral analysis”. The research investigated the characteristics of multi-parameter water quality detection spectral signals and background interferences, the main factors contributing to their formation, and potential solutions. A denoising method combining Ensemble Empirical Mode Decomposition (EEMD) and Singular Value Decomposition (SVD) was proposed, incorporating an improved universal threshold filtering technique. The proposed EEMD-SVD denoising method demonstrated excellent noise reduction performance in actual spectral data, further proving its effectiveness and superiority.

The VDMA tool was used to convert the Convolutional Neural Network (CNN) model into a hardware implementation code, enabling its deployment and operation on hardware platforms such as Field Programmable Gate Arrays (FPGAs). A multi-parameter online water quality detection system based on ZYNQ (Zynq-7000 SOC chip, equipped with a dual-core ARM Cortex-A9 processor) was developed, achieving rapid and accurate detection of heavy metal ion concentrations.

Based on the ZYNQ heavy metal ion water quality detection system, and in conjunction with the research and analysis of relevant national and industry standards and regulations, six water samples with copper ion concentrations were randomly selected. The study results showed that all measured relative errors were less than 7.32% and did not exceed 10%, indicating good accuracy. The repeatability test results were less than 4.06%, below 10%, demonstrating the system’s good reproducibility. The test results validated the reliability and feasibility of the application of the water quality detection system, with all key technical indicators meeting the national environmental protection standards and satisfying the application requirements for on-site multi-parameter water quality detection.

## Figures and Tables

**Table 1:** Number of neural network parameters.

Type/Step Length	Nuclear Shape	Number of Parameters
convolution/1	3 × 3 × 3 × 64	576
convolution/1	64 × 3 × 3 × 3 × 64	36,864
max pooling/1	2 × 2	
convolution/1	64 × 3 × 3 × 128	73,728
convolution/1	128 × 3 × 3 × 128	147,456
max pooling/2	2 × 2	
convolution/1	128 × 3 × 3 × 256	294,912
convolution/1	256 × 3 × 3 × 256	589,824
convolution/1	256 × 3 × 3 × 256	589,824
max pooling/2	2 × 2	
convolution/1	256 × 3 × 3 × 512	1,179,648
convolution/1	512 × 3 × 3 × 512	2,359,296
convolution/1	512 × 3 × 3 × 512	2,359,296
max pooling/2	2 × 2	
convolution/1	512 × 3 × 3 × 512	2,359,296
convolution/1	512 × 3 × 3 × 512	2,359,296
convolution/1	512 × 3 × 3 × 512	2,359,296
max pooling/2	2 × 2	
fully connected layer	25088 × 4096	102,760,448
fully connected layer	4096 × 4096	16,777,216
fully connected layer	4096 × 1	4096
Total number of parameters	134,251,072	

**Table 2:** Detection accuracy of concentrations of five metal solutions.

Metal Solutions	Cr	Hg	Pb	As	Cu
Accuracy (%)	90.707	96.676	96.113	90.909	99.394

**Table 3:** Names, models, and specifications of components in the monochromator.

Name	Model	Specification
Diaphragm	SK12	Φ1.0–12 mm
Plano-convex lens	General analytical optical quartz plain convex lens	Φ30 mm, f39.2 mm
Diffraction grating (Holographic)	GS012 Round Ball Technology	1200-line/mm, 20 mm × 20 mm × 2 mm
Lens carrier	Hengyang Optical MLNR-1.2	Φ30 mm
Optical bench	Kopu 25009	55 mm × 42 mm × 55 mm

**Table 4:** Standard curve for copper solution: analysis of its slope, intercept, and correlation coefficients.

Indicator	R	G	B	H	S	V	Grayscale Value
Intercept	0.18496	0.02133	−0.01037	−0.0431	−0.04644	−0.00164	0.03605
Slope	0.68115	0.53183	0.26334	0.32752	0.35757	0.28092	0.46806
Correlation coefficient	0.77405	0.91826	0.93965	0.76279	0.78905	0.93833	0.94095

**Table 5:** Comparison test experimental results for copper (Cu).

Test Object	Sample Number	Spectral Method (mg/L)	National Standard Method (mg/L)	Relative Error (%)	Repeatability (%)	Spectral Method Mean (mg/L)	National Standard Method Mean (mg/L)
1	1	0.082	0.084	−2.38%	1.84%	0.08	0.08
	2	0.079	0.082	−3.66%			
	3	0.079	0.082	−3.66%			
	4	0.08	0.083	−3.61%			
	5	0.081	0.083	−2.41%			
	6	0.078	0.08	−2.50%			
2	1	0.21	0.22	−4.55%	4.06%	0.22	0.23
	2	0.22	0.24	−8.33%			
	3	0.22	0.23	−4.35%			
	4	0.23	0.24	−4.17%			
	5	0.21	0.23	−8.70%			
	6	0.23	0.23	0.00%			
3	1	0.42	0.41	2.44%	2.28%	0.43	0.42
	2	0.44	0.43	2.33%			
	3	0.42	0.41	2.44%			
	4	0.44	0.43	2.33%			
	5	0.44	0.41	7.32%			
	6	0.43	0.41	4.88%			
4	1	0.55	0.52	5.77%	1.50%	0.54	0.53
	2	0.55	0.53	3.77%			
	3	0.54	0.52	3.85%			
	4	0.54	0.53	1.89%			
	5	0.55	0.52	5.77%			
	6	0.53	0.53	0.00%			
5	1	0.74	0.75	−1.33%	1.03%	0.73	0.75
	2	0.72	0.74	−2.70%			
	3	0.73	0.73	0.00%			
	4	0.73	0.76	−3.95%			
	5	0.73	0.75	−2.67%			
	6	0.72	0.75	−4.00%			
6	1	0.93	0.93	0.00%	1.46%	0.95	0.92
	2	0.93	0.93	0.00%			
	3	0.94	0.91	3.30%			
	4	0.95	0.92	3.26%			
	5	0.96	0.93	3.23%			
	6	0.96	0.92	4.35%			

## Data Availability

The original contributions presented in the study are included in the article; further inquiries can be directed to the corresponding author.
